# Advances in pediatric acute kidney injury pharmacology and nutrition: a report from the 26th Acute Disease Quality Initiative (ADQI) consensus conference

**DOI:** 10.1007/s00467-023-06178-4

**Published:** 2023-10-25

**Authors:** Molly Wong Vega, Michelle C. Starr, Patrick D. Brophy, Prasad Devarajan, Danielle E. Soranno, Ayse Akcan-Arikan, Rajit Basu, Stuart L. Goldstein, Jennifer R. Charlton, Erin Barreto, the ADQI 26 workgroup

**Affiliations:** 1 Renal and Apheresis Services, Texas Children’s Hospital, Houston, TX USA; 2 Department of Pediatrics, Division of Nephrology, Indiana University School of Medicine, Indianapolis, IN USA; 3 Pediatric and Adolescent Comparative Effectiveness Research, Department of Pediatrics, Indiana University School of Medicine, Indianapolis, IN USA; 4 Department of Pediatrics, Golisano Children’s Hospital, University of Rochester, Rochester, NY USA; 5 Division of Nephrology and Hypertension, Department of Pediatrics, Cincinnati Children’s Hospital Medical Center, University of Cincinnati, Cincinnati, OH USA; 6 Department of Bioengineering, Purdue University, West Lafayette, IN USA; 7 Divisions of Critical Care and Nephrology, Department of Pediatrics, Baylor College of Medicine, Texas Children’s Hospital, Houston, TX USA; 8 Division of Critical Care, Department of Pediatrics, Northwestern University, Chicago, IL USA; 9 Division of Nephrology, Department of Pediatrics, University of Virginia, Box 800386, Charlottesville, VA 22901 USA; 10 Department of Pharmacy, Mayo Clinic, Rochester, MN USA

**Keywords:** Acute kidney injury, Pharmacology, Nutrition, Pediatrics, Neonates, Development

## Abstract

**Background:**

In the past decade, there have been substantial advances in our understanding of pediatric AKI. Despite this progress, large gaps remain in our understanding of pharmacology and nutritional therapy in pediatric AKI.

**Methods:**

During the 26th Acute Disease Quality Initiative (ADQI) Consensus Conference, a multidisciplinary group of experts reviewed the evidence and used a modified Delphi process to achieve consensus on recommendations for gaps and advances in care for pharmacologic and nutritional management of pediatric AKI. The current evidence as well as gaps and opportunities were discussed, and recommendations were summarized.

**Results:**

Two consensus statements were developed. (1) High-value, kidney-eliminated medications should be selected for a detailed characterization of their pharmacokinetics, pharmacodynamics, and pharmaco-“omics” in sick children across the developmental continuum. This will allow for the optimization of real-time modeling with the goal of improving patient care. Nephrotoxin stewardship will be identified as an organizational priority and supported with necessary resources and infrastructure. (2) Patient-centered outcomes (functional status, quality of life, and optimal growth and development) must drive targeted nutritional interventions to optimize short- and long-term nutrition. Measures of acute and chronic changes of anthropometrics, body composition, physical function, and metabolic control should be incorporated into nutritional assessments.

**Conclusions:**

Neonates and children have unique metabolic and growth parameters compared to adult patients. Strategic investments in multidisciplinary translational research efforts are required to fill the knowledge gaps in nutritional requirements and pharmacological best practices for children with or at risk for AKI.

## Introduction

The 26th Acute Disease Quality Initiative (ADQI) served as a call to action for comprehensive care in the treatment of pediatric acute kidney injury (AKI) and disease. While in the last decade there have been significant advances in our understanding of pediatric AKI management and therapeutics, the roles of pharmacology and nutrition have been largely neglected. This ADQI meeting acknowledged the essential and unique roles pharmacotherapy and nutrition therapy play in treating children with AKI and disease or those who are at risk for developing AKI.

Pharmacotherapeutics and nutrition are disciplines which are inextricably linked in pediatric patients and share common challenges. Serum creatinine is the most common metric to assess kidney function for medication management and is impacted by nutritional status. Disease states and medications that can contribute to AKI such as shock and vasoactive drugs may alter regional blood flow and therefore nutrient utilization. Furthermore, both pharmacology and nutrition have been neglected topics of pediatric AKI research. As an example, research into the optimal method to dose medication and nutrition therapy is limited. Standard antibiotic doses used in pediatric critically ill children may lead to subtherapeutic or supratherapeutic exposure [1]. Therapeutic drug monitoring of medications is rare and developmentally tailored dosing guidance is limited [2]. Hospitalized children with AKI experience the highest rates of energy and protein deficits [3]. The resultant high prevalence of malnutrition is associated with substantial mortality in children with AKI [4]. Suboptimal nutritional management may contribute to high rates of muscle protein breakdown, metabolic derangements, poor fluid and electrolyte stewardship, and reduced rehabilitative capabilities necessary for survivors. The growth and developmental challenges that underlie every aspect of targeted medication and nutrition therapy in pediatric patients, particularly those with AKI and acute kidney disease (AKD), are understudied.

Herein, we address these inter-related topics by focusing on the specific challenges faced while caring for children with AKI, and propose future research goals to provide optimal individualized care.

## Methods

The 26th ADQI Consensus Conference, the first ADQI devoted to pediatric AKI, was held in Napa, CA, USA, in November 2021, as described [5]. The consensus-building process was informed by review of articles by workgroup members identified through a PubMed search. A modified Delphi method was used to reach consensus, using evidence-based literature, whenever possible. A summary of the consensus conference has been published [5]. Within this article, we provide a summary of the current knowledge of pharmacotherapy and nutritional therapy in pediatric AKI and the existing knowledge gaps, and detail recommendations for next steps.

## Results

Critical recommendations of the panel focused on a need to improve our understanding and treatment of (1) pharmacotherapy and (2) nutrition therapy in pediatric AKI by addressing the significant knowledge gaps therein (Table 1). These consensus recommendations are as follows:

- *Pharmacotherapy*: High-value, kidney-eliminated medications should be selected for a detailed characterization of their pharmacokinetics, pharmacodynamics, and pharmaco-“omics” in sick children across the developmental continuum. This will allow for the optimization of real-time modeling with the goal of improving patient care. Nephrotoxin stewardship will be identified as an organizational priority and supported with necessary resources and infrastructure.
- *Nutrition therapy*: Patient-centered outcomes (functional status, quality of life, and optimal growth and development) must drive targeted nutritional interventions to optimize short- and long-term nutrition. Measures of acute and chronic changes of anthropometrics, body composition, physical function, and metabolic control should be incorporated into nutritional assessments.

**Table 1:** Consensus summary of gaps, questions and recommendations for research agenda in pharmacology of pediatric acute kidney injury

Gap	Questions	Research agenda
Medication management	What is the optimal method for safely and effectively dosing medication in critically ill pediatric patients?	1. Characterize the impact of AKI on non-kidney drug clearance pathways in pediatric patients, superimposed on developmental changes 2. Refine models for characterizing the impact of developmental maturation on pharmacokinetics in pediatric critically ill patients 3. Determine best practices for post-marketing research on neonatal and pediatric pharmacology in the critically ill with and without AKI 4. Prioritize drugs and outcomes that are most important for a direct (non-extrapolated) pharmacokinetic evaluation in the pediatric and neonatal population
Nephrotoxin burden	How do we determine the appropriate level of nephrotoxin exposure for a patient?	1. Determine the drug specific factors which confer intensity of risk 2. Develop standards for nephrotoxin monitoring, including development of standardized practices and monitoring
Pharmacovigilance	What is best practice to ensure the safe and effective use of nephrotoxins in critically ill pediatric patients with or at risk for AKI?	1. Empirically test the KDIGO bundle in this population 2. What is the role for artificial intelligence in pharmacovigilance evaluations? 3. How does kidney replacement therapy alter pharmacokinetics?

To fully address the current state of evidence leading this consensus recommendation and opportunities for future research advancement, we sought to answer the two questions developed during the ADQI Consensus Conference.

### Question 1: What actions promote the safe and effective use of kidney eliminated and nephrotoxic medications in children?

#### Pharmacokinetic changes in critically ill pediatric patients have been understudied

Critically ill patients experience changes in the absorption, distribution, metabolism, and elimination of medications [6]. While enteral administration of medications is often the preferred route, factors may affect the adequacy of absorption including changes in gastric pH, decreased perfusion, delayed gastric emptying or dysmotility, bowel compromise, or altered anatomy. Subcutaneous, intramuscular, and transdermal absorption may be altered by perfusion changes and subcutaneous edema, both of which are common in critical illness. For these reasons, intravenously administered drugs offer the most predictable therapeutic effect in the critically ill. Vascular permeability in shock and heart failure may expand volume of distribution and decrease the plasma concentrations of drugs and minerals [7]. Altered protein binding from vascular permeability, decreased protein synthesis, and catabolism change the free fraction of drugs. Metabolism and elimination through the liver may be affected by altered blood flow or enzymatic activity. Kidney function may be increased or decreased in critical illness which affects drug elimination [8]. Disease states common in critical illness, including sepsis and shock, are associated with an increased risk of AKI. A proportion of critically ill patients experience the opposite problem—supraphysiologic kidney function—so-called “augmented renal clearance” which has been linked to decreased drug exposure and treatment failure in adults [9]. However, there has been limited investigation into this syndrome in pediatric patients. Some of these pharmacokinetic changes are obvious (e.g., decreased albumin concentration suggesting altered protein binding) whereas others are assumed, but not readily verified (e.g., changes to liver enzymatic activity).

#### AKI further changes pharmacokinetics

AKI can modify both pharmacokinetics and pharmacodynamics. While drug elimination via the kidney is altered, so too are other aspects of the pharmacokinetic profile including the volume of distribution, plasma protein binding, and non-kidney metabolism. Patients with AKI often experience an expanded volume of distribution of hydrophilic drugs due to the increase in total body water [6]. Fluid overload is common in critically ill patients and impacts drug dosing [10]. Although newer technology is available to directly assess blood volume, its viability for the pediatric and neonatal population is uncertain, and rarely is the tool used clinically to tailor pharmacotherapy. In severe AKI, the presence of uremic molecules may displace drugs from protein bindings sites.

AKI may also impact non-kidney elimination pathways. Experimental models have identified alterations to metabolism in the presence of AKI, but these have been insufficiently corroborated in humans. Studies also report decreased activity of transporter proteins (such as organic anion transporters in the proximal tubule) which could lead to increased drug exposure [11]. All of these challenges can be further accentuated when patients are on any form of continuous kidney replacement therapy (CKRT), where the kinetics of medication and nutrition therapy are rarely studied [12, 13]. In patients on CKRT, dosage, timing, and duration of medications merit careful attention and are areas in need of further investigation.

#### Extrapolating drug dosing in pediatric patients

Little is known about optimal dosing of medication and nutrition therapy for hospitalized pediatric patients. Drugs are mainly designed for and tested in adults, and mostly in non-critically ill patients. Pediatric extrapolation of medication and nutrition therapy dosing was proposed and supported to an extent by regulatory agencies based on three underlying assumptions: (1) similar disease progressions in the two populations, (2) similar responses to interventions, and (3) similar exposure–response relationships [14]. However, this decision-making was based on several decades old literature and clinical practice. As our technology has improved and our care has become more sophisticated, the knowledge gap has widened. For example, the physiology and pharmacokinetics of an extremely premature 24-week neonate weighing 500 g were not anticipated as part of the pediatric drug dosing extrapolation. Additionally, developmental changes in drug clearance are not accounted for in the weight-based dose recommendations [15].

Most data extrapolated from adult studies may not be appropriate for this patient population, especially during critical illness. Pediatric dosing is based on a decision tree model, asking if drugs have (1) similar progression and/or responses, and (2) similar exposure and/or response [14, 16]. To improve the efficiency of pharmacology studies in infants and children, opportunistic sampling and sparse sampling, noninvasive matrices (e.g., hair, exhaled breath, saliva), and microvolume assays have been suggested [17, 18]. In opportunistic and sparse sampling methods, a single procedure is performed to limit the burden of phlebotomy for research. This may include alignment of specimen collection with standard of care procedures (e.g., blood draw), or recovery of residual specimens drawn for clinical care for research purposes. These techniques limit the burden of research both for the patients and for the families, and for the research community, a priority of great importance in the pediatric and neonatal populations. Drawing the right balance between sufficient sampling and patient burden, including low recruitment, to clinical studies is one of the challenges faced in this field.

#### Developmental changes impact drug disposition and response

Developmental changes enhance complexity to the perturbed pharmacokinetics in critical illness [19]. Across the continuum of pediatric patients, body composition, organ size, organ function, and gene expression vary, leading to alterations in cellular function and metabolic activity. For example, total body water changes dramatically in the first months of life, from 85% in neonates to 60% by 6 months of age. This change is accompanied by increases in protein mass and muscle [20]. Furthermore, some tissues may be more sensitive to effects of medication early in life. Drug metabolizing enzyme activity and alterations in kidney function also evolve over time [1].

Evaluating drug disposition in critically ill pediatric patients, especially neonates, requires a comprehensive assessment of developmental factors that augment pharmacokinetic complexity. Estimating kidney function in neonates with critical illness is poorly described [21]. Although kidney function develops with maturation, the biomarkers utilized for kidney assessment have inadequate performance in neonates, especially those who are premature or critically ill [22]. In the first days of life, neonatal serum creatinine, the traditional mainstay of endogenous filtration markers, reflects maternal serum creatinine. As kidney function matures after birth, serum creatinine slowly declines and GFR increases [23]. Neonates also have less muscle, and therefore less creatinine production, which needs to be accounted for in GFR estimation [15]. Accurately tracking an infant on this dynamic curve of GFR maturation is not yet achievable at the bedside [24]. Furthermore, premature neonates may have ongoing post-natal nephrogenesis, but glomerulogenesis may be abnormal in this population [25].

#### Nephrotoxin burden is large in pediatric patients

Nephrotoxin exposure is a significant predictor of AKI in pediatric and neonatal patients in both the hospital and community [26, 27]. Up to one-fourth of the medications administered to hospitalized patients may be nephrotoxic and can result in potentially avoidable adverse drug events [28]. The odds of AKI in pediatric patients increased when exposed to nephrotoxins (OR 1.7, 95% CI 1.04–2.9) [26]. While it is both impractical, and likely inappropriate, to eliminate the use of all nephrotoxic therapies, the benefit must be weighed against the risk.

Nephrotoxin exposure is uniquely challenging in pediatrics given the biologic variable of development on the risk for drug toxicity. Developmental factors may affect AKI risk in neonates, as a potentiator or a mediator. An individual’s glomerular filtration rate gradually increases from birth, reaching adult values at approximately 2 years of age. During this transition, the risk of nephrotoxicity may be increased, particularly with substances eliminated by the kidney [23]. In neonates, the immaturity of the tubules in early life may play a role in the mechanism for nephrotoxins with injury that increases with increasing intracellular concentration (e.g., aminoglycosides) [19]. Concurrently, a neonate’s inability to maximally concentrate their urine may predispose to intravascular volume depletion which increases the nephrotoxic potential of medications impacting kidney hemodynamics [23]. Sex differences have been seen in nephrotoxin-associated AKI [29]. These may need to be accounted for with the hormonal changes that occur postnatally, and throughout childhood and adolescence.

Nephrotoxin burden, defined as the cumulative or aggregate exposure to nephrotoxins, is a major risk factor in pediatric AKI [30]. Both the duration of drug exposure and the nephrotoxic potential of the drug influence overall burden. Risk is potentiated with exposure to multiple nephrotoxins simultaneously [26]. Exposure to multiple nephrotoxins is common in neonates with critical illness, especially those born prematurely [31]. Studies have shown that the risk of AKI increases with use of simultaneous nephrotoxins, with those exposed to three or more medications being at greater risk of developing higher stage AKI than those with lower levels of exposure [26]. In a French pharmacovigilance study, concomitant administration of ≥ 2 nephrotoxins was associated with 66% of AKI cases [32]. In adults, each additional nephrotoxin used in the post-AKI period is associated with a 1.3-fold higher risk of new or progressive CKD even after adjustment for relevant confounders. For individuals with > 4 nephrotoxins, there is a 2.1-fold higher risk of CKD in 3 years [33]. Nephrotoxic risk is often cumulative, and pediatric patients (especially critically ill children and neonates) are at high risk of exposure to multiple nephrotoxins simultaneously. Evidence of long-term risk for CKD is beginning to emerge [34].

#### Pharmacovigilance and nephrotoxin stewardship programs are necessary to improve outcomes in pediatrics

Best practices for decreasing the incidence, severity, and complications associated with nephrotoxin exposure, particularly when multiple nephrotoxins are used concurrently, are still evolving. While standardized approaches to pediatric patients have been developed [35], and the suggested KDIGO bundle includes nephrotoxin stewardship [36], neither of these approaches have been formally tested for benefit in children. Stewardship in general integrates key principles including individual and institutional commitment, data, resources, and tools including to facilitate implementation of best practices. One pediatric-specific model of nephrotoxin stewardship is the NINJA (Nephrotoxic Injury Negated by Just in Time Action) program. In patients at high risk for nephrotoxic medication-associated AKI, daily serum creatinine monitoring is recommended [37]. With this relatively simple intervention, there was a 25% reduction in AKI rate and 42% reduction in AKI days per 100 nephrotoxin medication exposure in the first year. This comes from rapid AKI recognition and nephrotoxic medication discontinuation. By 3 years, the results were sustained with a 31% AKI intensity decrease and a 64% AKI rate decrease. These nephrotoxin programs have been expanded to neonates, and demonstrated decreased risk of nephrotoxic medication exposure while maintaining a high serum creatinine surveillance rate [31]. Importantly for the smallest neonates, the Baby NINJA program did not increase the need for blood transfusions [38]. Further potential extensions include developing reflex serum creatinine monitoring for high-risk patients and local medication list tailoring [39].

The success of the nephrotoxin stewardship programs depends on engaged physicians, supportive hospital leadership, active discussion, and multidisciplinary engagement with physicians and pharmacists [40]. Sustained QI requires a culture change throughout the multidisciplinary team. Despite growing evidence that quality programs are effective at improving nephrotoxin stewardship in pediatric patients, best practices for dissemination and implementation of these programs require further characterization, especially in neonates.

#### Research recommendations to address existing gaps

Despite a recent growth in our understanding of pharmacokinetics and pharmacodynamics, there remain large gaps in our understanding (Table 1). We recommend that high-value, kidney-eliminated medications should be selected for a detailed characterization of their pharmacokinetics, pharmacodynamics, and pharmaco-“omics” in sick children across the developmental continuum, to allow optimization of real-time modeling towards developing improvements in patient care. Selection of these medications will necessarily be determined by the frequency of use, therapeutic window, and context of the evaluation. As an example, a detailed assessment of a frequently used kidney-eliminated chemotherapy agent in sick children could provide substantial benefits for safety and effectiveness. Another case is the development of a deeper understanding of the pharmacology of kidney eliminated anti-epileptics will help with therapeutic tailoring over a child’s development. Furthermore, we recommend that nephrotoxin stewardship should be identified as an organizational priority and supported with necessary resources and infrastructure.

### Question 2: What are the nutritional targets in children with acute kidney injury and disease and how are they defined?

#### Optimal nutritional management works in concert with pharmacologic management

Malnutrition and protein energy wasting is common in hospitalized children with AKI, with a prevalence rate of 30–55% in children receiving CKRT [4, 41–43]. Nutritional management may prevent worsening nutritional status in children with AKI, persistent AKI, and AKD. Inability to provide adequate nutrition has often been cited as a significant factor in the decision to initiate CKRT in the pediatric population. Initiation of CKRT can improve fluid management and may prevent worsening nutritional status [3, 44]. However, there are few studies which evaluate the effect of nutritional therapy on outcomes beyond mortality.

The Pediatric Renal Nutrition Taskforce, an international collaborative of pediatric dietitians and nephrologists, recently published clinical practice recommendations for the nutritional management of children with AKI [45]. These recommendations were supported by an extensive literature review and Delphi process. Moreover, they were designed to provide clarity to the prescription of nutrition therapies and improve patient outcomes in children with AKI. Optimal nutritional management allows for control of metabolic derangements, control of fluid balance, and supported recovery of tissue repair and immune function. Despite the potential that nutritional stewardship holds, there is scarce evidence to support nutrition interventions to improve outcomes in children with AKI/AKD. Fluid overload has an independent and synergistic effect on the outcomes of critically ill children with AKI [46]. Nutritional fluids are modifiable and can be optimized to improve fluid control prior to worsening AKI while positively influencing the frequency of metabolic abnormalities in children with AKI. These clinical practice recommendations work in concert with the research recommendations to help identify key areas needed for advancement of nutritional therapy in children AKI/AKD.

#### Age-related nutrient requirements impact nutrition risk

The dynamic nature of AKI, AKD, and recovery within the developmental and growth spectrum of a pediatric patient limits the accuracy of extrapolative nutrition interventions in clinical practice. Malnutrition and protein energy wasting has been independently associated with mortality in children receiving CKRT [4]. Yet, mortality is a crude end point measurement as malnutrition has multiple contributory mechanisms and outcomes that may be bidirectionally confounded by AKI medical management and dialytic modalities. The effect of age and its influence on developmental stage needs to be considered as malnutrition disproportionally affects younger children [47].

#### AKI and critical illness affect the metabolic response and goals to nutrition therapy

The physiological targets and goals for nutritional management change based on the severity of AKI/AKD, nutritional status, and age. Acutely, nutritional targets should focus on normalization of electrolytes and minerals as well as volume control through proper stewardship of nutritional fluids. Once these initial targets are achieved, the patient’s therapeutic nutrition goals should shift towards supporting rehabilitative processes including promoting improved metabolic function, immune function, tissue repair, and mitigating lean mass loss and functional decline. In addition to these nutritional targets, it is critical to understand the effects of AKI/AKD on nutritional status. Key nutritional principles to consider in this paradigm are included in Table 2.

**Table 2:** Contributors to nutrition imbalance in children with AKI

Mechanism [69]	Common contributors to nutrition imbalance [70, 71]
Decreased intake	Iatrogenic Fluid management Conservative management
Malabsorption	Feeding modality (enteral vs parenteral) Formula composition (hydrolyzed vs. polymeric) Tube placement site Gastrointestinal disturbances Metabolic alterations in AKI
Increased losses	Dialytic modality and losses Gastrointestinal disturbances
Hypermetabolism	Dialysis Inflammation/metabolic alterations in AKI Nitrogen balance Metabolic changes in energy expenditure
Altered nutrient utilization	Dialytic modality Medication contribution to nutrition/macronutrients Dialysis solutions Feeding modality Metabolic and nutrient kinetic alterations Medication related substrate Inflammation

#### Nutrient kinetics and medication exposure alter macronutrient need in children with AKI

Specific macronutrient targets are extremely limited as no calorimetric or nutrient kinetic studies have been reported on children with AKI. Catabolism compounded with nutritional deficits are frequently reported in children [48] and adults [49] with AKI. Historically, CKRT was considered a contraindication to indirect calorimetry given potential influence of bicarbonate clearance. Data support a potential underestimation of energy expenditure in adults and thus inappropriate prescription of energy [50]. Additionally, extrapolation from both critically ill pediatric [51] and adult AKI [52] data confirms a shift in substrate utilization due to inflammatory driven metabolic dysfunction resulting in increased beta oxidation.

Medication and dialysis fluid exposure compound non-nutritional energy and substrate exposure, most notably in the form of dextrose and citrate-based anticoagulation, leading to the equivalent of 500 additional calories daily in adults [53]. Net calorie loss can also occur if utilizing glucose free replacement solutions [53]. Excessive or suboptimal provision of energy and substrate may lead to prolonged recovery time. The need for individualized energy prescriptions may hinge on the technological advancements required to make indirect calorimetry a more utilized tool in hospitalized children. Additional research should focus on utilizing indirect calorimetry or other techniques, such as doubly labeled water, with careful emphasis on the need to study the effect of dialysis and medication-related fluid exposure in children with AKI.

Baseline protein requirements are higher in children than in adults given the need for development and growth. Increased muscle protein breakdown in AKI/AKD drives negative nitrogen balances [54]. Attenuation of skeletal muscle loss through adequate nutrition support is critical in supporting tissue repair, rehabilitation, and improved functional outcomes in survivors. In acute intermittent dialytic therapies and conservative management, protein needs are often extrapolated from CKD or maintenance dialytic therapy yet steady-state energy and protein metabolism are primary assumptions that should not be assumed in acute or critical illness. The metabolic consequences of underfeeding may also subsequently lead to increased catabolism due to redirection of protein as a substrate for energy.

More work is needed to understand each child’s unique nutritional needs in the setting of AKI. Pediatric CKRT studies consistently report losses equivalent to ~ 10–20% of amino acids provided via nutrition support [41, 55]. Most amino acid concentrations appear to stabilize after 5 days of initiation of CKRT [55]. However, carnitine, a key nutrient in long-chain fatty acid metabolism, has been reported as a critical amino acid of concern in children receiving CKRT with secondary carnitine deficiency reported in 100% of patients by the third week of CKRT [56]. Secondary carnitine deficiency has the potential to negatively influence rehabilitative processes given its effects on cardiac and skeletal muscle. Thus, specific dosing targets of protein or specific amino acids in children with AKI and AKD remain undetermined, but should be dosed based on nutrition-related outcome metrics.

#### Critical illness and AKI further modify electrolyte, mineral, vitamin, and trace element requirements

Optimal management of micronutrients in children with AKI and AKD is even more elusive. Electrolyte abnormalities are hallmark complications of AKI, but are dynamic and dependent on the child’s clinical status, medications, and CKRT-dialytic modality. The most commonly reported mineral abnormalities in children receiving CKRT are hypophosphatemia and hypomagnesemia [57]. Adequate electrolyte and mineral control may have long-term rehabilitation effects. Improved phosphate levels (an essential requirement for energy production) in adults receiving CKRT is associated with fewer ventilator days [58]. Studies reporting nutritional intake and AKI rarely report electrolyte or mineral intakes from nutrition *and* pharmacological sources. Therapeutic targets for electrolytes and minerals should be adjusted based on monitoring levels.

Little is known also regarding vitamin and trace element needs in children with AKI and AKD. There have been two studies of micronutrient balance in children receiving CKRT which evaluated folate, selenium, zinc, copper, chromium, and manganese, and found negative balances of folate and selenium were potentially present within the first week of CKRT [55, 59]. Vitamin C has received less attention despite its water soluble nature and likelihood of losses in effluent [60]. Recent studies have raised the question if CKRT, AKI, or critical illness contributes to larger variation in plasma levels of micronutrients in metabolic disturbances. It is also possible that CKRT duration and primary diagnosis (e.g., burns, multi-organ failure) pose the greater risk for micronutrient-deficient states [61].

Lastly, there is limited evidence that micronutrient supplementation improves patient outcomes in *any* hospitalized child. Micronutrient monitoring is not standard practice and often not attained until overt physical manifestations of a deficiency are present. Laboratory values of most micronutrients can be affected by inflammation, movement between plasma and intracellular compartments, decreased sequestration, and increased exposure to reactive oxygen species [62]. The ideal circumstance would be a baseline assessment of micronutrient values before critical illness and a trend over time, particularly in patients with prolonged illness. Rarely will this be available; thus, differentiating between the physiological response to illness versus deficient states becomes extremely difficult. Ideally, future research regarding micronutrient needs, intake, and outcomes likely lies with a multidisciplinary approach to identify the appropriate provision of micronutrients. An integrative approach is needed, encompassing pre-clinical, epidemiological, and translational models including well-designed prospective studies, particularly evaluating micronutrient metabolism changes and balance during periods of high oxidative stress and inflammation.

#### Feeding modality may mitigate adverse outcomes but requires further study

Enteral feeding is recommended in critically ill children in order to maintain gut integrity and mitigate gut atrophy. Enteral protein intake has been associated with lower 60-day mortality compared to parenteral (PN) amino acids in critically ill children [63]. In adults receiving CKRT, low protein intake has been associated with increased mortality, which is primarily delivered enterally [49]. This association of protein intake supporting improved mortality has not been found in children with AKI. Most studies analyzing protein losses in CKRT are in children receiving PN in which clearance and metabolic dysfunction due to development and growth may not be similar [41, 55]. Additionally, the PEPaNIC (early versus late parenteral nutrition in the pediatric ICU) trial in critically ill children showed that withholding PN for the first week of an ICU admission decreased the incidence of new infections and improved time to recovery when compared to supplemental PN [64]. In early PN, the dose of amino acids was associated with increased infections and longer dependency on mechanical ventilation for critically ill children [65].

It is theorized that in the acute phase of critical illness, catabolism may be part of a larger adaptive and beneficial process of autophagy which may initially be protective. However, enteral nutrition has been reported as a barrier to achieving nutritional goals in children receiving CKRT [66]. High rates of gastrointestinal dysmotility in critically ill children with AKI compared to those without AKI likely limit the ability to initiate or advance enteral feeding [67]. Thus, PN may be necessary to achieve nutrition targets in children with AKI, especially those who are malnourished. The nutritional effects of feeding the gut are likely much more nuanced and need further investigation.

#### Advancement of nutritional therapy relies on identification of appropriate outcomes

The principles of nutrition therapy in children with AKI, persistent AKI and AKD, should promote nutrient balance to support normalization, recovery, and rehabilitation in survivors. Thus, outcome metrics should reflect these end goals to allow for appropriate comparisons of nutritional interventions. An evaluation of nutritional intervention efficacy is also dependent on our ability to accurately diagnose phenotypic nutritional morbidities (e.g., malnutrition, kidney cachexia, frailty, and sarcopenia). Lastly, it is difficult to adequately identify nutritional outcome metrics when follow-up in AKI survivorship in pediatrics is scarce. Nutrition support and mobilization have been shown to attenuate muscle loss and promote muscle protein synthesis in critically ill children [68]. AKI PICU survivors have high rates of poor functional outcomes and experience increased technological dependence after hospital discharge [43]. Ideally, monitoring variables should include those that would optimize the rehabilitative process, allowing for the recuperation of functional status, improvement in lean mass and muscle strength, promotion of muscle protein synthesis, and reversal of metabolic alterations such as insulin resistance (Table 3).

**Table 3:** Proposed nutrition-related outcomes [69, 70]

Anthropometrics: * –appropriately adjusted for volume status* Growth Nutritional phenotype
Body composition: Fat mass Intramuscular fat deposition Lean mass
Functional status: Muscle strength Cardiorespiratory fitness Functional status/ability to perform ADLs Technology dependence Developmental delay/decline (cognitive, physical) Quality of life
Immune dysfunction: Infections
Metabolic control: Electrolyte/mineral abnormalities Insulin resistance/hyperglycemia Beta oxidation/hypertriglyceridemia * Inflammatory cascade*
Delayed recovery: Tissue repair AKI/kidney recovery end points
Nutritional Balance “intake vs need”: Macronutrients * –assessed with medication related intake & measured metabolic rate* Micronutrients Electrolyte

#### Research recommendations to address existing gaps

Patient-centered outcomes such as functional status, quality of life, and optimal growth and development must drive the targeted nutritional interventions, optimizing short- and long-term nutrition, incorporating measures of acute and chronic changes of anthropometrics, body composition, physical function, and metabolic control (Table 4).

**Table 4:** Consensus summary of gaps, questions, and recommendations for research agenda in nutrition of pediatric acute kidney injury

Nutrition management	What is the optimal method for safely and effectively delivering nutrition to critically ill pediatric patients?	1. Develop and test the suitability of specific diagnostic tools to guide real-time decision making for medication and nutrition therapy 2. Determine the acute and chronic factors that influence the dosing of medication and nutrition therapy in critically ill pediatric patients
Nutritional dosing	What are the principles of nutritional management in AKI/AKD?	1. Identify key nutrition scientists/dietitian stake holders to support research in pediatric AKI/AKD 2. Promote and advocate for clinical dietitian training and education in critical care nephrology, quality improvement and research
	What are the therapeutic nutritional targets and goals for in children with AKI and AKD?	1. Integrate dietitians/nutrition scientists in development of collaborative databases for observational cohorts 2. Determine optimal research designs to analyze metabolic changes in pediatric AKI 3. Evaluate resource availability to accurately perform metabolic studies in pediatric AKI 4. Develop multi-center collaborative nutrition intervention RCTs
Monitoring of nutrition related outcome metrics	What metrics should be followed to meet therapeutic nutritional targets in children with AKI/AKD?	1. Identify relevant nutritional related outcome metrics important for children with AKI and AKD

## Conclusion

With advancing CKRT technologies and as the boundaries of viability have been pushed in the neonatal intensive care unit, pharmacology and nutrition in pediatric AKI have been understudied. Further research advancements in the field of pediatric AKI must focus on the unique aspects of development as a biological variable, a detailed characterization of high value, kidney-eliminated medications, and patient-centered outcomes to drive targeted nutritional interventions.
